# Supplementary material for: Metabolomic Profiles in Starved Light Breed Horses during the Refeeding Process
Source: Animals (Basel). 2022 Sep 21;12(19):2527. doi: 10.3390/ani12192527 (PMC9559287; doi:10.3390/ani12192527)
Supplement: Supplementary file 1 [file animals-12-02527-s001.zip › animals-1892616-supplementary.pdf]

Metabolomic Profiles in Starved Light Breed Horses During the Refeeding Process

Sawyer C. Main [1], Lindsay P. Brown [2,*], Kelly R. Melvin [1], Shawn R. Campagna [2,3], Brynn H. Voy [1,4], Hector F. Castro [2,3], Lewrell G. Strickland [1,5], Melissa T. Hines [5], Robert D. Jacobs [6], Mary E. Gordon [6] and Jennie L. Z. Ivey [1,*]

[1] Department of Animal Science, University of Tennessee, 2506 River Drive, Knoxville, TN 37996, USA
[2] Department of Chemistry, University of Tennessee, 1420 Circle Drive, Knoxville, TN 37996, USA
[3] Biological and Small Molecule Mass Spectrometry Core, University of Tennessee, 1416 Circle Drive, Knoxville, TN 37996, USA
[4] Department of Nutrition, University of Tennessee, 1215 W. Cumberland Ave., Knoxville, TN 37996, USA
[5] College of Veterinary Medicine, University of Tennessee, 2407 River Drive, Knoxville, TN 37996, USA
[6] Purina Animal Nutrition, 100 Danforth Drive, Gray Summit, MO 63039, USA
* Correspondence: lbrow121@vols.utk.edu (L.P.B.); jzivey@utk.edu (J.L.Z.I.); Tel.: +1-865-974-3141 (L.P.B.); +1-865-974-3157 (J.L.Z.I.)

Table S1. Metaboanalyst results from T-Test comparisons between CCP and RP for blood chemistries (plasma)

Analyte	t.stat	p.value	FDR
CREAT	-2.0819	0.04051	0.28524
ALB	-2.0757	0.041096	0.28524
LDH	-1.899	0.061123	0.28524
AST	-1.6905	0.094774	0.33171

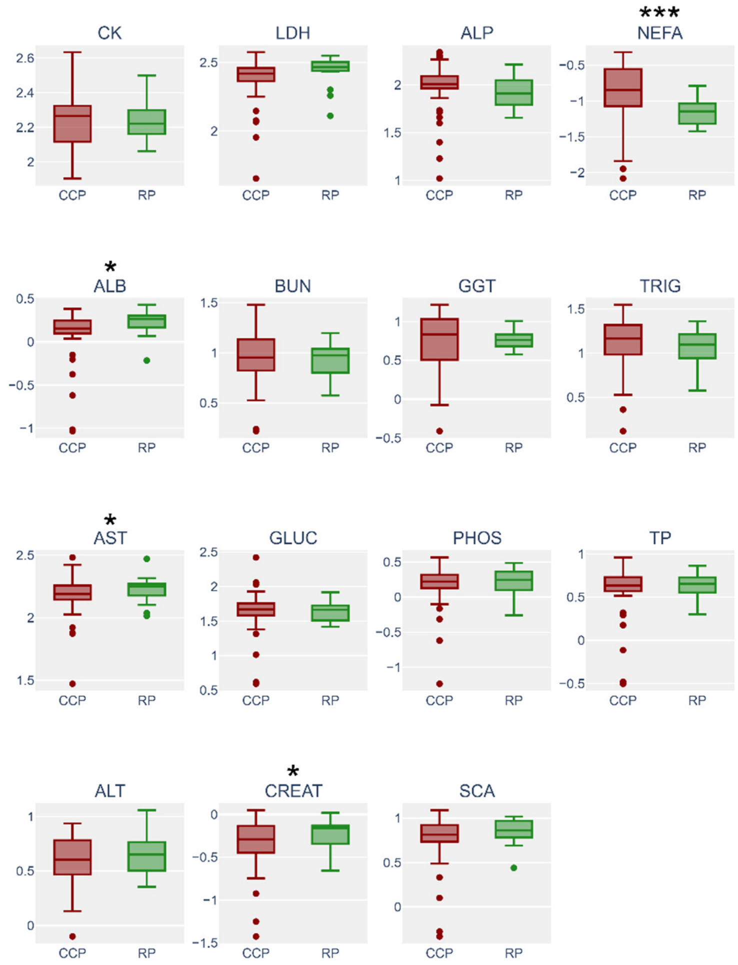

Figure S1. Box plots of blood serum data conveying the differences between the CCP and RP. Asterisks represent statistical significance (p value ≤ 0.1 = *; p value ≤ 0.05 = **; p value ≤ 0.01 = ***). LDH: lactate dehydrogenase (p value 0.07), U/L; ALP: alkaline phosphatase, U/L; ALB: albumin (p value 0.06), g/dL; BUN: blood urea nitrogen, mg/dL; GGT: gamma-glutamyl transferase, U/L; AST: aspartate amino transferase, U/L; TP: total protein, g/dL; GLUC: glucose, mg/dL; PHOS: phosphate, mg/dL; ALT: alanine transaminase, U/L; CREAT: creatinine (p value 0.08), mg/dL; TRIG: triglycerides, mg/dL; CK: creatinine kinase, U/L; NEFA: non-esterified fatty acids (p value 0.00_6), mEq/L; SCA: serum calcium, mg/dL

Table S2. Metaboanalyst results from T-Test comparisons between diet (C vs. S) for blood chemistries (plasma)

Analyte	t.stat	p.value	FDR
BUN	-3.1391	0.002364	0.033102
Phos	-2.7717	0.006916	0.048412
TP	-2.3217	0.022765	0.10624
ALB	-1.9277	0.057404	0.16412
GLUC	-1.8552	0.067205	0.16412
LDH	-1.8339	0.070336	0.16412

Table S3. Metaboanalyst results from T-Test comparisons between sex (mare vs. gelding) for blood chemistries (plasma)

Analyte	t.stat	p.value	FDR
CREAT	2.8082	0.006241	0.047123
Phos	2.7092	0.008228	0.047123
ALB	2.6343	0.010098	0.047123

Table S4. Metaboanalyst results from T-Test comparisons between age group (below 20 vs. 20 and above) for blood chemistries (plasma)

Analyte	t.stat	p.value	FDR
Phos	-3.7581	0.000322	0.004503
ALB	-3.1685	0.002162	0.015134
LDH	-2.4968	0.01456	0.067946
TP	-2.0713	0.041513	0.14528
ALT	1.9732	0.051884	0.14528
BUN	-1.7888	0.077386	0.18057

Table S5. Metaboanalyst results from T-Test comparisons between diet (C vs. S) for blood chemistries (serum)

Analyte	t.stat	p.value	FDR
BUN	-2.5498	0.012687	0.10881
PHOS	-2.5235	0.013601	0.10881
TP	-2.1816	0.032076	0.17107
ALT	2.026	0.0461	0.1844
SCA	-1.867	0.065564	0.2098
ALB	-1.7397	0.085757	0.22868

Table S6. Metaboanalyst results from T-Test comparisons between sex (mare vs. gelding) for blood chemistries (serum)

Analyte	t.stat	p.value	FDR
PHOS	3.1301	0.00244	0.022982
ALB	3.0758	0.002873	0.022982
CREAT	2.9336	0.00437	0.023309
TRIG	2.2569	0.026744	0.10698
SCA	1.8891	0.062502	0.20001
CK	1.6753	0.097773	0.22348

Table S7. Metaboanalyst results from T-Test comparisons between age group (below 20 vs. 20 and above) for blood chemistries (serum)

Analyte	t.stat	p.value	FDR
PHOS	-3.6473	0.00047	0.007525
ALT	3.194	0.002008	0.012152
ALB	-3.1526	0.002279	0.012152
SCA	-2.1607	0.033709	0.11742
TP	-2.0545	0.043193	0.11742
CK	-1.978	0.051371	0.11742
LDH	-1.7817	0.078587	0.15717

Table S8. Fold change analysis for RP compared to CCP (RP/CCP) for metabolomics data

Metabolite	Fold change (RP/CCP)	p.value
alpha-Ketoglutarate	0.724568	0.196767
Uric acid	0.598083	0.078713
Indoleacrylate	0.631762	0.2454
Allantoin	0.372438	0.009124
Uridine	0.542886	0.036214
S-Adenosyl-L-methioninamine	0.97674	0.93986
Xylose	0.770332	0.115634
Taurine	0.668531	0.197207
Arginine	0.892785	0.491743
Tryptophan	1.045125	0.743274
Serine	0.774025	0.214143
Creatine	0.295335	0.001054
Citrulline	1.166122	0.518447
Alanine/Sarcosine	0.461614	0.002627
Valine/betaine	0.687569	0.201204
Tyrosine	0.828572	0.362614
Proline	0.888966	0.530212
Phenylalanine	0.681895	0.098501
Ornithine	1.056064	0.83341
Cystine	0.988447	0.96667
N-Acetylornithine	3.842559	0.000283
Methionine	0.545381	0.03917
Homoserine/Threonine	0.774824	0.297029
Glutamine	0.611119	0.085949
Cystathionine	0.777079	0.10732
Creatinine	0.911952	0.63489

Table S9. Metaboanalyst results from T-Test comparisons between diet (C vs. S) for metabolites

Metabolite	t.stat	p.value	FDR
N-Acetylornithine	3.5499	0.000628	0.14928
254.1395	-3.3856	0.001072	0.14928
Taurine	3.3077	0.001374	0.14928
380.2437	-3.2342	0.001732	0.14928
199.1335	-3.1841	0.002022	0.14928
243.1234	-3.1719	0.0021	0.14928
Glutamine	3.1629	0.002159	0.14928
261.1339	-3.1014	0.002605	0.15759
Cystine	3.0386	0.003148	0.16928
449.2626	-2.9641	0.003926	0.19004
Citrulline	2.9155	0.004528	0.19922
459.3049	-2.8684	0.005189	0.20929
S-Adenosyl-L-methioninamine	2.835	0.005712	0.21265
Ornithine	2.799	0.006328	0.21338
414.3332	-2.7264	0.007758	0.21338
211.1336	-2.6854	0.008692	0.21338
Methionine	2.6707	0.00905	0.21338
336.1812	2.661	0.009296	0.21338
326.2331	2.647	0.009659	0.21338
Homoserine/Threonine	2.6369	0.009927	0.21338
238.1445	2.623	0.01031	0.21338
361.2703	2.6217	0.010346	0.21338
432.2746	-2.616	0.010508	0.21338
384.2386	-2.6134	0.010581	0.21338
270.2072	-2.5864	0.011381	0.21515
216.1600	-2.5745	0.011749	0.21515
243.1233	-2.5655	0.012035	0.21515
Uridine	2.5486	0.012591	0.21515
252.1602	-2.5397	0.012891	0.21515
329.2159	-2.5107	0.013922	0.22232
142.1234	-2.5021	0.014239	0.22232
271.2103	-2.4569	0.016025	0.23216
Valine/betaine	2.4472	0.016431	0.23216
alpha-Ketoglutarate	2.4397	0.016753	0.23216
242.1112	-2.424	0.017448	0.23216
229.1633	-2.4113	0.018026	0.23216
369.2747	2.4044	0.018348	0.23216
356.2436	-2.3987	0.018614	0.23216
252.1237	-2.3968	0.018707	0.23216
408.2749	-2.3444	0.021363	0.25257
356.2435	-2.336	0.021816	0.25257
Serine	2.3268	0.022325	0.25257

286.2018	-2.3236	0.022506	0.25257
501.3041	-2.3006	0.023836	0.25257
222.1493	-2.2982	0.023978	0.25257
268.1913	-2.2977	0.024005	0.25257
413.2650	2.2855	0.024741	0.25395
220.0975	2.2783	0.025185	0.25395
186.1495	-2.2439	0.027405	0.26287
264.2157	-2.2356	0.027967	0.26287
466.2911	-2.229	0.02842	0.26287
151.0873	-2.2189	0.029128	0.26287
188.1006	-2.2177	0.02921	0.26287
202.1081	-2.2113	0.029667	0.26287
209.0040	-2.2048	0.030139	0.26287
305.1754	-2.201	0.030415	0.26287
217.0293	2.1636	0.033268	0.28249
369.2392	-2.1363	0.035496	0.29566
477.3249	-2.1299	0.036041	0.29566
392.2436	-2.1171	0.037137	0.29957
140.0714	-2.0935	0.039254	0.30601
209.1542	-2.0773	0.040759	0.30601
191.0711	-2.0773	0.04076	0.30601
Alanine/Sarcosine	2.074	0.041073	0.30601
306.1787	-2.0705	0.0414	0.30601
116.9722	-2.0639	0.042039	0.30601
251.1523	-2.0549	0.04292	0.30601
307.1547	2.0542	0.042994	0.30601
374.9682	2.0362	0.044809	0.30867
175.0858	-2.0327	0.045165	0.30867
215.1284	-2.0239	0.046083	0.30867
270.1342	-2.0239	0.046088	0.30867
322.2015	-2.0169	0.046826	0.30867
125.7532	-2.0135	0.047193	0.30867
146.8665	-2.0066	0.047928	0.3093
334.2381	-1.9782	0.051112	0.32114
369.1158	1.972	0.051823	0.32114
307.1581	1.9697	0.052093	0.32114
360.2578	-1.9669	0.052417	0.32114
429.2963	-1.9457	0.054958	0.32884
598.4149	-1.9451	0.055034	0.32884
360.9761	1.9339	0.056414	0.33056
450.2857	-1.9261	0.057392	0.33056
283.1029	-1.9236	0.057707	0.33056
230.1756	-1.9192	0.058277	0.33056
278.1756	-1.9156	0.058736	0.33056
182.0818	-1.9032	0.060365	0.33392

485.3228	1.8945	0.061516	0.33392
xylose	1.8919	0.061863	0.33392
309.2179	-1.8902	0.062093	0.33392
228.1600	-1.8728	0.064488	0.34163
358.2423	-1.867	0.065312	0.34163
446.2907	-1.8646	0.065644	0.34163
305.2117	-1.8416	0.068974	0.35514
248.9599	-1.83	0.070719	0.35523
383.3270	-1.8259	0.071336	0.35523
Indoleacrylate	1.8233	0.07173	0.35523
627.5379	-1.8182	0.072519	0.35523
242.1757	-1.8173	0.072661	0.35523
320.2224	1.8114	0.073566	0.35606
170.0431	1.8027	0.074937	0.35664
Phenylalanine	1.7967	0.0759	0.35664
399.2857	-1.7789	0.078797	0.35664
119.0347	-1.7776	0.079007	0.35664
241.1079	-1.7762	0.079235	0.35664
328.2124	1.7745	0.079512	0.35664
238.1081	-1.7744	0.079538	0.35664
448.2698	-1.7741	0.079581	0.35664
169.0866	1.7632	0.081417	0.3614
302.1966	-1.759	0.082137	0.3614
250.1809	-1.7359	0.086168	0.37278
318.2108	-1.7353	0.086264	0.37278
338.2332	-1.7261	0.087917	0.37562
334.2019	-1.7076	0.091327	0.37562
370.2785	-1.7037	0.092046	0.37562
135.0813	-1.701	0.092548	0.37562
312.2174	-1.6988	0.092981	0.37562
113.0970	-1.6911	0.094435	0.37562
255.1234	-1.689	0.094848	0.37562
370.2589	-1.6875	0.09514	0.37562
434.2542	-1.6855	0.095511	0.37562
Allantoin	1.6844	0.095732	0.37562
418.2593	-1.6805	0.096497	0.37562
231.1790	-1.678	0.09698	0.37562
371.2545	-1.6757	0.097422	0.37562
381.2105	-1.6739	0.097785	0.37562

Table S10. Metaboanalyst results from T-Test comparisons between sex (mare vs. gelding) for metabolites

Metabolite	t.stat	p.value	FDR
Creatine	-5.9958	4.63E-08	2.24E-05
Cystine	-5.7997	1.08E-07	2.61E-05
Alanine/Sarcosine	-5.5842	2.69E-07	3.81E-05
Taurine	-5.547	3.15E-07	3.81E-05
Serine	-5.3606	6.87E-07	5.91E-05
Allantoin	-5.3451	7.32E-07	5.91E-05
Glutamine	-5.286	9.35E-07	6.46E-05
165.128	5.1739	1.48E-06	8.95E-05
Valine/betaine	-5.142	1.69E-06	9.07E-05
Phenylalanine	-4.7807	7.15E-06	0.000346
alpha-Ketoglutarate	-4.5786	1.57E-05	0.00069
S-Adenosyl-L-methioninamine	-4.4384	2.67E-05	0.001079
213.1322	4.2656	5.10E-05	0.001836
Homoserine/Threonine	-4.2546	5.31E-05	0.001836
Uridine	-4.2359	5.69E-05	0.001836
Tryptophan	-4.1614	7.47E-05	0.00226
Indoleacrylate	-4.1338	8.26E-05	0.002352
xylose	-4.0584	0.000108	0.002916
434.2542	3.8803	0.000204	0.005192
Cystathionine	-3.8212	0.00025	0.005779
Tyrosine	-3.8207	0.000251	0.005779
Ornithine	-3.7736	0.000295	0.006492
Methionine	-3.6992	0.00038	0.008005
198.1018	3.5296	0.000671	0.012935
188.1006	3.5254	0.00068	0.012935
330.2281	3.519	0.000695	0.012935
762.5618	3.5059	0.000726	0.013007
Citrulline	-3.4081	0.000997	0.017238
230.1545	3.3378	0.001249	0.020815
229.1633	3.3277	0.00129	0.020815
334.2020	3.3095	0.001367	0.02134
128.1079	3.2725	0.001536	0.023228
236.1652	3.2016	0.001916	0.028105
278.1756	3.1544	0.002216	0.031543
501.3041	3.1256	0.00242	0.033461
383.2779	3.0857	0.002731	0.036498
285.2177	3.0743	0.002827	0.036498
261.1339	3.0605	0.002947	0.036498
254.1395	3.0601	0.002951	0.036498
292.1745	3.0486	0.003054	0.036498
202.1081	3.0446	0.003092	0.036498
414.3332	2.9629	0.00394	0.045405
242.1757	2.954	0.004045	0.045526
292.1914	2.9366	0.004257	0.046823

305.1754	2.9216	0.004447	0.04747
311.2050	2.9167	0.004512	0.04747
177.1328	2.8571	0.005361	0.055202
Creatinine	-2.8446	0.005557	0.055519
360.2385	-2.8406	0.005621	0.055519
199.0972	2.8309	0.005778	0.055927
459.3049	2.8075	0.006176	0.058613
264.1318	2.791	0.006472	0.060243
326.1969	2.7521	0.007221	0.065279
269.2118	2.749	0.007283	0.065279
271.2104	2.7397	0.007476	0.065788
395.1682	2.7325	0.007628	0.065925
295.0518	2.7152	0.008004	0.067183
257.1392	2.7131	0.008051	0.067183
306.1787	2.6971	0.008417	0.069045
270.2072	2.6862	0.008672	0.069957
177.0597	2.6555	0.009436	0.074872
231.1790	2.6447	0.009717	0.075857
313.2130	2.6237	0.010289	0.079049
175.0858	2.6078	0.010744	0.081248
116.9722	2.5868	0.011367	0.084638
343.2594	-2.5781	0.011637	0.085335
203.1115	2.5371	0.01298	0.093768
382.2746	2.5238	0.013446	0.095703
252.1237	2.5161	0.013723	0.096259
135.0813	2.5021	0.01424	0.098459
386.2910	2.4314	0.017115	0.11667
200.1288	2.4136	0.017919	0.12045
268.1913	2.4015	0.018483	0.12255
302.2332	2.394	0.018841	0.12296
322.2383	2.3896	0.019054	0.12296
175.1165	2.3784	0.019605	0.12485
422.2905	2.3731	0.01987	0.1249
277.1439	2.3291	0.022196	0.13543
398.2652	2.3274	0.022293	0.13543
202.1526	2.3258	0.022385	0.13543
248.9599	2.3145	0.023024	0.13758
137.0605	2.3009	0.023816	0.13996
Uric acid	-2.2978	0.024001	0.13996
293.0486	2.2905	0.024442	0.14083
141.0166	2.2775	0.025239	0.14371
248.1651	2.2689	0.02578	0.1445
184.1702	2.2658	0.025975	0.1445
409.2419	-2.2509	0.026943	0.14819
441.3327	-2.2168	0.029276	0.15921
227.1479	2.2024	0.030313	0.16249
242.1112	2.198	0.030634	0.16249
228.1600	2.1946	0.030886	0.16249
87.04483	-2.1819	0.031847	0.16574

236.2016	2.1737	0.032477	0.16722
243.1233	2.1554	0.033921	0.17163
600.5022	2.154	0.034042	0.17163
446.2907	2.139	0.035271	0.17599
232.0134	-2.126	0.036373	0.17964
386.2693	2.1128	0.037519	0.18343
463.3170	2.0963	0.03899	0.18786
466.2911	2.094	0.039203	0.18786
448.2698	2.0789	0.040607	0.19061
225.1492	2.0758	0.040899	0.19061
411.2857	2.0752	0.040958	0.19061
262.1807	2.0474	0.043667	0.20128
196.0612	2.0406	0.044354	0.20252
127.0010	2.0287	0.045582	0.20619
305.2117	2.0088	0.047693	0.21374
271.2103	1.9911	0.049641	0.22043
299.2053	1.9784	0.05109	0.22452
253.1441	1.9749	0.05149	0.22452
200.0536	1.9662	0.052501	0.22688
321.0795	1.9553	0.053792	0.2281
181.1231	1.9469	0.054805	0.2281
204.0914	1.9439	0.055178	0.2281
281.1863	-1.943	0.055291	0.2281
461.3097	1.9426	0.055332	0.2281
238.1081	1.9404	0.055611	0.2281
202.0119	1.9337	0.056444	0.22957
263.1285	1.9211	0.058027	0.23404
339.1809	1.9106	0.059382	0.23753
187.0972	1.8881	0.062389	0.24751
356.2435	1.8838	0.062977	0.24781
322.2015	1.8795	0.063567	0.24812
145.0140	1.8686	0.065086	0.25113
293.1867	1.8569	0.066754	0.25113
284.2053	-1.8565	0.066811	0.25113
322.1322	1.8517	0.067502	0.25113
214.1443	1.8497	0.067798	0.25113
315.1922	1.8487	0.067944	0.25113
294.2067	-1.8441	0.068609	0.25113
369.1158	1.8412	0.069035	0.25113
252.1602	1.8397	0.069265	0.25113
434.2542	1.8379	0.069527	0.25113
598.4149	1.8252	0.071438	0.25612
209.1542	1.8182	0.072515	0.25689
399.2857	1.8165	0.072771	0.25689
212.1652	1.8127	0.073364	0.25689
284.1498	1.8101	0.073778	0.25689
146.8665	1.806	0.07441	0.25724
329.2159	1.7804	0.078534	0.26865
194.9461	1.7759	0.079281	0.26865

238.1445	1.7754	0.079374	0.26865
266.1509	1.7435	0.084821	0.28346
87.04486	1.7429	0.084922	0.28346
278.1473	1.7386	0.085682	0.28404
370.2785	1.735	0.086325	0.28423
156.9904	1.7264	0.087858	0.28732
292.1551	1.7208	0.088879	0.28756
230.1756	1.7195	0.089121	0.28756
248.2016	1.7092	0.091019	0.29174
229.1439	-1.6991	0.092919	0.29393
370.2589	-1.6973	0.093247	0.29393
327.2366	1.6959	0.093524	0.29393
397.2702	1.69	0.094657	0.29557
361.2014	1.675	0.097564	0.3027

Table S11. Metaboanalyst results from T-Test comparisons between age group (below 20 vs. 20 and above) for metabolites

Metabolite	t.stat	p.value	FDR
Creatine	4.8452	5.55E-06	0.002685
Alanine/Sarcosine	3.5068	0.000723	0.17503
181.1231	-2.7932	0.006432	0.94452
Methionine	2.3907	0.019	0.94452
Allantoin	2.3768	0.019682	0.94452
135.0813	-2.3649	0.020287	0.94452
87.04483	2.2813	0.025001	0.94452
285.1258	2.1792	0.032048	0.94452
238.1445	2.0992	0.038733	0.94452
Uridine	2.0911	0.039469	0.94452
250.1809	2.0403	0.044385	0.94452
Homoserine/Threonine	1.9935	0.049377	0.94452
202.1081	-1.9614	0.053073	0.94452
248.2016	-1.9523	0.05415	0.94452
279.2435	1.9481	0.054662	0.94452
397.2702	-1.9272	0.057254	0.94452
293.1754	-1.9191	0.058289	0.94452
Indoleacrylate	1.9185	0.058369	0.94452
380.2437	-1.9146	0.058869	0.94452
199.0972	1.9143	0.058904	0.94452
294.2067	-1.8887	0.062307	0.94452
alpha-Ketoglutarate	1.853	0.067316	0.94452
360.2385	1.8411	0.069059	0.94452
277.1805	1.8285	0.070937	0.94452
283.1029	-1.8083	0.074053	0.94452
162.965	-1.7913	0.076758	0.94452
292.1914	-1.7761	0.079248	0.94452
231.1790	-1.7611	0.081783	0.94452
204.0914	-1.7586	0.082207	0.94452
125.7532	-1.7512	0.083474	0.94452
250.1444	-1.7423	0.085038	0.94452
514.3166	1.7407	0.085315	0.94452
223.1335	-1.7371	0.085958	0.94452
N-Acetylornithine	-1.7341	0.086487	0.94452
299.2048	1.7182	0.089361	0.94452
330.2281	-1.6951	0.093683	0.94452
271.2103	-1.6888	0.094873	0.94452
311.2224	-1.6809	0.096407	0.94452
Tyrosine	1.6773	0.097109	0.94452

Table S12. Metaboanalyst results from PLS-DA VIP comparisons between diet (C vs. S) metabolomic profiles

Metabolite	Comp. 1	Comp. 2	Comp. 3	Comp. 4	Comp. 5
Allantoin	4.9501	3.4884	3.2075	3.1004	3.033
Creatine	4.8721	3.4723	3.1908	3.0151	2.9872
Taurine	4.8263	3.4319	3.1531	2.9823	2.914
Indoleacrylate	4.7862	3.6691	3.3831	3.2227	3.1766
S-Adenosyl-L-methioninamine	4.7471	4.0184	3.6936	3.4939	3.4125
Cystine	4.5001	3.1606	2.9134	2.784	2.7274
Valine/betaine	4.4518	3.2091	2.9628	2.7975	2.7346
alpha-Ketoglutarate	4.0085	3.1742	2.9163	2.766	2.699
Uridine	3.8664	3.4838	3.2065	3.0297	2.956
Glutamine	3.8059	2.7835	2.5578	2.4153	2.3603
Phenylalanine	3.535	2.6187	2.4116	2.277	2.2217
Alanine/Sarcosine	3.438	2.4322	2.2347	2.1242	2.0727
Serine	3.1169	2.2314	2.0503	1.9698	1.9236
Ornithine	3.0492	3.1175	2.8683	2.7095	2.6437
Methionine	3.0301	3.214	2.9647	2.7989	2.7305
Homoserine/Threonine	2.8964	2.4632	2.2638	2.1485	2.0962
Tryptophan	2.835	2.1139	2.0102	1.9509	1.9282
194.9461	2.8027	2.6944	3.7282	3.5238	3.5877
463.3170	2.7781	2.011	1.9204	1.937	1.8989
Tyrosine	2.7355	2.5918	2.3815	2.2817	2.2261
214.1443	2.6734	3.0823	3.0728	3.3626	3.3273
Uric acid	2.5831	4.6735	4.3362	4.0947	3.9965
Cystathionine	2.5711	2.2546	2.0714	1.9564	1.9098
Citrulline	2.4915	2.8205	2.5942	2.4495	2.3904
395.1682	2.4507	2.8571	2.6653	2.7535	2.7142
Creatinine	2.3691	3.3959	3.1291	2.9691	2.8971
xylose	2.2443	2.0368	1.872	1.7821	1.7405
322.2015	2.027	2.4769	2.4224	2.313	2.4962
284.2053	2.0061	2.6226	2.4244	2.5722	2.5262
321.0795	1.8318	1.994	1.9013	2.2998	2.2627
299.2048	1.8256	1.3645	1.3763	1.335	2.183
295.0518	1.7991	1.8614	2.1115	2.0306	1.9843
477.3249	1.6924	1.1894	1.1017	1.2197	1.1916
292.1745	1.6447	1.7441	1.6752	1.6095	1.6193
441.3327	1.6379	1.5059	1.4983	1.4342	1.4195
343.2594	1.6062	1.4851	1.3769	1.3693	1.376
204.0914	1.6029	1.8377	2.048	1.9345	1.9329
434.2542	1.5656	1.7131	1.5808	1.4925	1.4585
326.2331	1.5202	2.1434	2.0129	1.9118	1.9364
237.1606	1.5126	1.7105	1.5715	1.4883	1.683
87.04483	1.5098	1.8048	1.6754	1.5994	1.5724
229.1439	1.5094	1.2294	1.3564	1.2816	1.2506
N-Acetylornithine	1.507	3.972	3.6522	3.5013	3.424
296.1342	1.4791	1.0602	2.4472	2.3665	2.3111

424.2334	1.4481	2.7872	2.7286	2.7146	2.6894
196.0612	1.4145	1.4946	1.4031	1.5087	1.5806
209.0040	1.3924	0.98275	0.90303	0.97889	1.055
360.2385	1.3767	1.7957	1.6721	1.5883	1.6
227.1479	1.3024	1.8885	1.8066	1.7583	1.7184
309.0675	1.2857	1.5789	2.1871	2.1521	2.1108
200.0536	1.2722	1.8829	2.043	1.9892	1.9453
422.2905	1.2542	1.7093	1.5764	1.498	1.5088
232.0134	1.2493	1.5029	1.4876	1.4064	1.3727
145.0140	1.2434	1.633	1.5393	1.4544	1.4223
202.0119	1.2332	1.0567	1.3939	1.4095	1.3761
217.0025	1.21	1.2793	1.5695	2.0248	1.9763
141.0166	1.1985	1.5218	1.4514	1.3843	1.3513
309.2177	1.1834	1.6768	1.5424	1.6344	1.6999
313.213	1.1788	0.88801	0.94457	0.93209	0.90931
394.2595	1.1691	0.87869	0.80776	0.89406	0.87237
248.9599	1.1363	0.85547	0.83697	0.79013	0.77153
127.0010	1.1241	0.92074	0.84595	0.87431	0.87469
209.1178	1.121	0.81248	0.74694	0.7117	0.69711
322.2383	1.114	0.8374	0.83815	0.81635	0.80987
250.1082	1.0935	1.5441	1.4187	1.3779	1.3552
299.2053	1.0894	1.0323	0.99137	0.94868	0.9839
283.1029	1.0796	0.96795	1.2585	1.2208	1.2154
143.0346	1.0151	1.5563	1.4793	1.448	1.468
292.1914	1.015	0.98244	0.90565	0.88066	0.88378
279.1631	1.0101	0.80698	0.74912	0.73298	0.7583

Table S13. Metaboanalyst results from PLS-DA VIP comparisons between sex (mare vs. gelding) metabolomic profiles

Metabolite	Comp. 1	Comp. 2	Comp. 3	Comp. 4	Comp. 5
N-Acetylornithine	4.6122	2.7464	2.6083	2.5508	2.4743
S-Adenosyl-L-methioninamine	4.5062	3.8014	3.4374	3.3589	3.2789
Taurine	4.4276	3.099	2.8106	2.7545	2.6736
477.3249	3.7725	2.5006	2.5979	2.5472	2.4868
Cystine	3.7042	2.8596	2.5938	2.5346	2.4733
119.0347	3.6513	2.497	2.4127	2.3909	2.3259
326.2331	3.5285	3.2147	3.1868	3.1552	3.0891
Glutamine	3.4776	2.5838	2.3349	2.2831	2.2219
Uridine	3.4568	3.297	2.9806	2.917	2.8339
Valine/betaine	3.2835	3.2054	2.8981	2.8327	2.7477
Ornithine	3.2767	2.7548	2.4914	2.4345	2.3752
alpha-Ketoglutarate	3.2292	3.0931	2.8172	2.7717	2.6885
Indoleacrylate	3.1791	4.0033	3.6177	3.5361	3.4389
Methionine	3.1725	2.7677	2.5456	2.5009	2.4255
209.0040	3.1425	4.315	4.2853	4.2282	4.1008
322.2015	3.04	2.3999	2.2063	2.2872	2.2402
Citrulline	3.0364	2.3858	2.1561	2.1097	2.0642
194.9461	2.9192	2.3321	3.1983	3.5322	3.4552
Homoserine/Threonine	2.663	2.3083	2.0955	2.0495	1.9937
169.0866	2.5269	2.3755	2.1607	2.1137	2.0508
Allantoin	2.4832	3.7456	3.4309	3.353	3.2783
155.0014	2.3423	1.5706	1.51	1.5454	1.5181
374.9682	2.3225	1.9613	1.7785	1.7602	1.715
336.1812	2.2992	2.4654	2.3842	2.3339	2.2687
Creatine	2.2026	3.0439	2.7927	2.7317	2.6537
Serine	2.1247	2.129	1.9373	1.8966	1.8487
538.9967	2.0828	1.5964	1.5852	1.6558	1.606
Phenylalanine	2.0569	2.8431	2.5872	2.5291	2.4533
Alanine/Sarcosine	2.0389	2.4413	2.2105	2.1642	2.1018
217.0293	2.0124	1.5461	1.4304	1.4255	1.4709
334.2019	1.8903	1.7472	1.6692	1.6388	1.6058
449.2626	1.8811	1.6719	1.7477	1.7627	1.7237
283.1029	1.8645	1.7141	1.5722	1.5366	1.6324
170.0431	1.8148	1.1521	1.043	1.1686	1.2328
315.0411	1.7889	1.0805	2.7849	2.7755	2.7513
238.1445	1.7881	1.5043	1.4592	1.4268	1.3854
279.1631	1.7544	1.2388	1.1196	1.1103	1.0941
Creatinine	1.7387	3.1456	2.8726	2.8177	2.7333
380.2437	1.7321	1.4209	1.2938	1.2644	1.2286
320.2224	1.7285	1.8757	1.7419	1.7026	1.6533
360.9761	1.6988	1.2662	1.1543	1.1457	1.1773
334.2381	1.6589	0.9963	0.9043	1.0228	1.0482
Tyrosine	1.6248	2.8504	2.5879	2.5305	2.4568

408.2749	1.6243	1.0836	0.98304	0.96066	0.94297
256.1600	1.5781	1.0323	1.0882	1.1884	1.3215
xylose	1.5686	2.095	1.903	1.8661	1.8107
209.1178	1.5571	0.95751	0.89185	0.88722	0.87904
214.1443	1.5302	3.2454	3.0311	2.9863	3.2695
369.2392	1.5015	1.4415	1.5275	1.4938	1.4526
355.2233	1.477	0.9411	0.86252	0.87475	0.86774
356.2435	1.4736	1.0805	0.97672	0.9564	0.92761
398.2686	1.4132	0.8541	0.77577	0.76413	0.74113
286.2018	1.403	1.0174	1.0373	1.016	0.99024
Arginine	1.3976	0.8356	0.87335	0.85932	0.84733
94.9249	1.3717	1.7353	1.7333	1.6977	1.7106
182.0818	1.3609	1.0429	1.0078	0.98536	0.9562
250.1809	1.3487	0.96793	0.89914	0.87862	0.85236
Tryptophan	1.332	2.6252	2.3751	2.3225	2.2556
141.0748	1.3215	1.042	0.95401	0.93354	1.0327
Cystathionine	1.3152	2.6717	2.4154	2.3616	2.2903
295.0518	1.2997	1.1225	1.0294	1.1905	1.3211
166.0174	1.2963	0.82538	1.1037	1.1013	1.6166
307.1547	1.2887	1.4103	1.4599	1.4313	1.3881
369.2747	1.2845	1.3925	1.37	1.3681	1.3276
248.9599	1.2734	0.75351	0.70985	0.74315	0.73527
485.3228	1.2656	1.1919	1.2109	1.2148	1.207
87.04485	1.2615	0.80875	1.391	1.4474	1.4764
201.1006	1.2447	0.73904	1.1097	1.0869	1.2637
309.2179	1.2188	1.0354	1.0245	1.0045	0.97922
252.9047	1.2053	0.72081	0.81513	0.85076	0.95009
145.0140	1.1686	1.042	1.0794	1.0552	1.0991
598.4149	1.1626	0.76085	0.68779	0.67233	0.65223
224.0229	1.1593	0.69138	0.67774	0.73596	0.95503
299.2048	1.1519	1.1716	1.0684	1.0468	1.2782
264.2157	1.1403	1.0759	1.0214	1.0019	0.97608
358.2423	1.1373	0.8818	0.80396	0.78561	0.7621
87.04487	1.1322	1.0606	0.96167	0.95096	0.93069
162.965	1.1222	0.73095	0.68748	0.69619	0.74118
146.8665	1.117	0.66184	0.61072	0.59729	0.64741
376.2965	1.1119	1.0723	1.0154	0.99911	1.0056
422.2905	1.108	1.3894	1.2603	1.2366	1.1995
300.2057	1.1067	1.0653	0.96338	0.9445	0.9471
220.0975	1.1031	1.288	1.2629	1.2612	1.2252
296.1342	1.0932	3.011	3.1844	3.2669	3.1915
361.2703	1.0879	1.0272	0.9569	0.94324	0.91532
87.04485	1.0855	0.77261	0.8228	0.81801	0.7951
200.0536	1.0481	0.84324	0.83847	0.89018	1.2022
315.1922	1.0391	0.68012	0.90106	0.88379	0.85868
271.2103	1.0327	0.66583	0.65403	0.64411	0.6275
441.3327	1.0293	0.67446	0.61239	0.61437	0.59589

399.3218	1.027	0.70108	0.64542	0.66769	0.64837
227.1479	1.0266	1.3631	1.2597	1.2322	1.1988
191.0711	1.0138	0.76485	0.69987	0.68429	0.73976
396.2747	1.0134	0.61509	0.55663	0.55663	0.55337
218.1183	1.0056	1.727	1.5622	1.5796	1.5385
355.2485	1.0005	0.86667	1.3004	1.3596	1.3509

Table S14. Metaboanalyst results from PLS-DA VIP comparisons between age group (below 20 vs. 20 and above) metabolomic profiles

Metabolite	Comp. 1	Comp. 2	Comp. 3	Comp. 4	Comp. 5
S-Adenosyl-L-methioninamine	5.445	4.0496	3.7989	3.5455	3.4216
Taurine	5.0164	3.5786	3.3539	3.1447	3.0314
Cystine	4.8323	3.4417	3.2265	3.0163	2.9069
N-Acetylornithine	4.1585	2.9657	2.827	2.6516	2.5685
Indoleacrylate	4.1357	3.8846	3.64	3.405	3.2877
Glutamine	4.1205	2.9452	2.7597	2.5785	2.4851
Valine/betaine	4.0921	3.223	3.02	2.8268	2.7248
Uridine	4.086	3.4327	3.2277	3.0126	2.9031
alpha-Ketoglutarate	3.9437	3.2077	3.0066	2.8149	2.7165
Ornithine	3.8582	2.9242	2.7407	2.558	2.4717
Methionine	3.7755	2.9383	2.7531	2.5852	2.4927
Uric acid	3.5274	3.5959	3.3955	3.4184	3.3549
Allantoin	3.5254	3.6762	3.5286	3.3022	3.1876
Citrulline	3.2921	2.545	2.3869	2.2315	2.1652
Homoserine/Threonine	3.2867	2.4637	2.3092	2.158	2.088
Creatine	3.0556	2.9816	2.849	2.6597	2.565
194.9461	3.0432	3.3114	4.3203	4.0326	4.0193
Serine	3.0153	2.2175	2.0798	1.9919	1.9203
Alanine/Sarcosine	2.8701	2.3786	2.2393	2.0935	2.0175
Creatinine	2.8224	2.9738	2.787	2.629	2.539
Phenylalanine	2.7477	2.8181	2.641	2.4714	2.3821
Tyrosine	2.4668	2.7718	2.6003	2.4309	2.3437
326.2331	2.4651	3.546	3.3289	3.2358	3.1576
477.3249	2.3723	1.8316	1.8528	1.9747	2.0985
xylose	2.2077	2.0528	1.9254	1.8065	1.7437
322.2015	2.194	2.5818	2.4322	2.4738	2.6208
119.0347	2.1166	1.7105	1.6058	1.6653	1.61
Tryptophan	2.0611	2.4767	2.322	2.1673	2.1102
299.2048	1.9299	1.379	1.3141	1.4401	1.5558
Cystathionine	1.8741	2.7465	2.5771	2.4053	2.3225
374.9682	1.8727	2.5293	2.4388	2.3304	2.2519
315.0411	1.6888	1.5087	1.8717	1.9391	2.2111
295.0518	1.6797	1.9585	1.9475	2.1568	2.0788
169.0866	1.6732	2.4199	2.2785	2.1373	2.1004
217.0293	1.642	1.9415	1.8222	1.7401	1.8112
Arginine	1.6099	1.2599	1.2365	1.1789	1.1481
283.1029	1.4611	1.8156	1.7046	1.7263	1.7682
441.3327	1.4343	1.1907	1.1962	1.1186	1.106

209.1178	1.4143	1.1904	1.1156	1.0447	1.0338
238.1445	1.3755	1.6677	1.6304	1.5968	1.544
369.2392	1.3528	2.0318	2.0405	1.9723	1.9122
237.1606	1.3476	1.3409	1.4723	1.5837	1.539
538.9967	1.33	1.4956	1.7762	1.666	1.6408
155.0014	1.3156	0.96587	1.0633	1.0718	1.053
380.2437	1.3055	1.5971	1.4993	1.4102	1.3596
408.2749	1.2937	1.1535	1.0839	1.055	1.0346
286.2018	1.2628	1.3441	1.3333	1.2752	1.2552
309.0675	1.2254	1.8438	2.0162	2.2583	2.2016
399.3218	1.2042	1.2757	1.1955	1.175	1.133
485.3228	1.198	1.78	1.7366	1.6419	1.6285
334.2381	1.1972	0.85517	0.80235	0.88246	1.132
395.1682	1.1914	0.94877	1.1244	1.2343	1.2608
449.2626	1.1891	1.3553	1.2982	1.3298	1.3272
336.1812	1.1882	2.0132	1.976	1.8559	1.8056
334.2019	1.1879	1.5683	1.5128	1.4139	1.4793
376.2965	1.1823	1.9729	1.8915	1.7807	1.8134
182.0818	1.1641	1.3286	1.2533	1.1833	1.1404
360.9761	1.1332	1.3087	1.3609	1.3213	1.3057
162.9650	1.0738	0.88868	0.86438	0.89114	0.86929
321.0795	1.0691	0.97485	1.0362	1.3647	2.007
204.0914	1.0683	1.2435	1.3395	1.4923	1.5794
322.2383	1.0542	0.77682	0.78596	0.73541	0.7638
309.2179	1.026	1.3366	1.3356	1.2903	1.2557
279.1631	1.0219	0.80418	0.75794	0.7375	0.7558
398.2686	1.0175	0.73318	0.69693	0.72276	0.71307
356.2435	1.0161	0.93123	0.87943	0.85109	0.82781
598.4149	1.011	0.93119	0.8794	0.82136	0.79514
399.1823	1.0072	0.76684	1.1087	1.6017	1.5692
